# Selective Laser Melting of Duplex Stainless Steel 2205: Effect of Post-Processing Heat Treatment on Microstructure, Mechanical Properties, and Corrosion Resistance

**DOI:** 10.3390/ma12152468

**Published:** 2019-08-02

**Authors:** Suvi Papula, Mingshi Song, Aaron Pateras, Xiao-Bo Chen, Milan Brandt, Mark Easton, Yuriy Yagodzinskyy, Iikka Virkkunen, Hannu Hänninen

**Affiliations:** 1 Centre for Additive Manufacturing, School of Engineering, RMIT University, Carlton, VIC 3053, Australia; 2 Discipline of Manufacturing, Materials and Mechatronics, School of Engineering, RMIT University, Carlton, VIC 3053, Australia; 3 Department of Mechanical Engineering, School of Engineering, Aalto University, P.O. Box 14200, FI-00076 Aalto, Finland

**Keywords:** duplex stainless steel, selective laser melting, heat treatment, microstructure, mechanical properties, residual stresses, corrosion resistance

## Abstract

Additive manufacturing (AM) is a rapidly growing field of technology. In order to increase the variety of metal alloys applicable for AM, selective laser melting (SLM) of duplex stainless steel 2205 powder and the resulting microstructure, density, mechanical properties, and corrosion resistance were investigated. An optimal set of processing parameters for producing high density (>99.9%) material was established. Various post-processing heat treatments were applied on the as-built predominantly ferritic material to achieve the desired dual-phase microstructure. Effects of annealing at temperatures of 950 °C, 1000 °C, 1050 °C, and 1100 °C on microstructure, crystallographic texture, and phase balance were examined. As a result of annealing, 40–46 vol.% of austenite phase was formed. Annealing decreased the high yield and tensile strength values of the as-built material, but significantly increased the ductility. Annealing also decreased the residual stresses in the material. Mechanical properties of the SLM-processed and heat-treated materials outperformed those of conventionally produced alloy counterparts. Using a scanning strategy with 66° rotation between layers decreased the strength of the crystallographic texture. Electrochemical cyclic potentiodynamic polarization testing in 0.6 M NaCl solution at room temperature showed that the heat treatment improved the pitting corrosion resistance of the as-built SLM-processed material.

## 1. Introduction

Materials performance is often a factor that limits available technological designs and solutions. Advances related to manufacturing methods and processes enable the development of high-performance metallic materials. Additive manufacturing (AM) technology offers many possibilities for localized microstructural control and material design [1,2,3,4]. Metal AM has grown significantly over the past decades and evolved into an industrial-scale manufacturing technique to enable the production of functional and structural components, of virtually any geometry, with high efficiency and accuracy. Selective laser melting (SLM), a powder bed fusion technique, is widely used for AM processing of metallic alloys.

The variety of metals currently used for AM processing is relatively limited [1,2]. SLM of stainless steels is, for a large part, utilizing austenitic stainless steels, most often AISI 316L. Application of SLM to austenitic-ferritic duplex stainless steels has been limited so far [5,6,7]. Duplex stainless steels combine many of the beneficial properties of both ferritic and austenitic steels. The yield strength of duplex stainless steels can be up to twice that of the single-phase austenitic and ferritic stainless steels, and they exhibit good toughness and excellent corrosion resistance. Their unique properties are dependent on the phase balance between face-centered cubic (FCC) austenite and body-centered cubic (BCC) ferrite. Davidson et al. [5] have studied the effect of SLM processing conditions and post-process heat treatment on the microstructure and properties of super-duplex stainless steel SAF 2507. The same material was also processed with SLM by Saeidi et al. [6], obtaining a fully ferritic microstructure with excellent mechanical properties, showing that the scanning strategy affects the microstructure, density, and macroscopic texture. Recently, Hengsbach et al. [7] published results on SLM-produced SAF 2205 (UNS S31803) duplex stainless steel, characterizing the microstructural features and phase balance after post-process heat treatments at various temperatures.

The mechanical properties—yield strength, in particular—of SLM-processed stainless steels can be considerably higher than that of the conventionally processed alloys with a similar chemical composition [6,8,9,10]. Unlike most strengthening mechanisms in crystalline materials, with simultaneous reduction in ductility, SLM can produce unique microstructural features in metals, leading to markedly enhanced strength and ductility [11]. This is attributed to the fast cooling rate, fine grain size, and, more importantly, the introduced dislocation network [12]. The very high dislocation density obtained in SLM processing has been explained by thermal contraction stresses during the fast solidification [8].

Due to the layer-upon-layer nature of the SLM-process, there is anisotropy in mechanical properties. The epitaxial grain growth during the solidification of the melt pools can result in highly textured microstructures. It has been shown that the microstructure of SLM-processed stainless steels (e.g., AISI 316L) characteristically show a high degree of anisotropy, with a strong {0 0 1} texture in the building direction [13,14]. Evolving crystallographic texture can be modified by varying the process parameters, such as laser power, laser scan speed, and layer thickness, because they affect the heat flow, thermal gradient, and extent of remelting of the previously deposited layers [15]. Altering the scanning strategy across the layers offers possibilities for controlling the formation of texture and anisotropy [2,16,17]. Scanning line stagger and rotation can alter the texture of SLM-processed parts by varying the directions of thermal gradients of successive layers. The crystallographic texture and growth direction of grains can have a significant effect on the resulting tensile properties of SLM-processed material [18].

Duplex stainless steels solidify initially as ferrite, and austenite phase starts to form at grain boundaries by solid state transformation during further cooling to between 1400 and 1200 °C. Additional austenite forms through a solid-state phase transformation during a subsequent heat treatment. Consequently, annealed duplex stainless steel has higher austenite volume fraction than in the as-cast or as-welded condition [19]. In earlier studies, it was reported that after SLM processing, the as-built microstructure of duplex stainless steels is almost fully ferritic [6,7]. Therefore, post-processing heat treatments are necessary for achieving a desired dual-phase microstructure. Post-processing heat treatment can also be beneficial for homogenizing and recrystallizing the as-built solidification structure and for relieving unfavorable residual stresses [20,21].

The unique, very fast thermal heating and cooling cycles during SLM processing introduce high residual stresses in the built material [21]. Thermal stresses occur due to the restricted expansion or contraction of the material in response to local temperature changes. Due to the layer-by-layer processing in SLM, most of the previously melted and solidified material layers experience a complex re-melting and re-solidification cycle. The formation of residual stresses in SLM processing can be described by two models, the temperature gradient mechanism and the cool-down mechanism [22]. Residual stresses introduced in a metallic sample during SLM processing are strongly affected by the heat input, scanning strategy, and other parameters. Decreasing the temperature gradient through preheating the building platform is the most common approach to reduce residual stresses [23].

In laser melting of stainless steels, careful control of microstructure is essential for achieving good corrosion resistance. The effects of processing on corrosion resistance of additively manufactured stainless steels have been rarely studied [24,25,26,27], and further research effort is required to gain an in-depth understanding of the topic. Due to the microstructural inhomogeneity and risk of porosity, caused e.g., by incomplete fusion between successive molten layers, SLM-processed stainless steel can be more susceptible to pitting corrosion than conventional bulk stainless steels, although their general corrosion resistance could be similar [28]. Localized defects can act as active sites for corrosion initiation, and microstructural heterogeneity can result in micro-galvanic corrosion. Then again, high cooling rates in SLM of stainless steels can, when properly controlled, result in a fine-grained microstructure with very little segregation, which improves their corrosion resistance [24,29]. Some published research results indicate that the pitting corrosion resistance of SLM-processed austenitic stainless steel AISI 316L can be even better than that of wrought AISI 316L, due to the absence of MnS inclusions in the microstructure of SLM 316L steel, provided that the porosity of the material is low (<1%) [30].

In order to increase the variety of metal alloys applicable for AM, this research concentrates on SLM of duplex stainless steel 2205 (UNS designation S31803), which is the most widely used duplex stainless steel, having high mechanical strength combined with excellent corrosion resistance, especially against stress corrosion cracking. This research aims to investigate the achievable microstructure, mechanical properties, corrosion resistance, and residual stresses in the as-built state and after various post-processing heat treatments.

## 2. Materials and Methods 

Pre-alloyed, gas-atomized powder of SAF 2205 (UNS S31803/EN 1.4462) duplex stainless steel, purchased from Sandvik Osprey Ltd., Neath, UK, was used in this study. The chemical composition of the powder is presented in Table 1.

**Figure 1:**
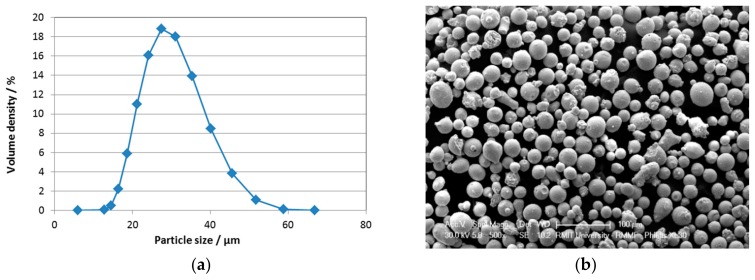
(**a**) Particle size distribution analyzed with laser particle sizing and (**b**) scanning electron microscopy (SEM) secondary electron image of the powder showing the particle morphology.

The quality of the metal powders has a marked effect on the quality of AM-processed final materials. The particle size distribution was analyzed with laser particle sizing using a Mastersizer 3000 equipment (Malvern Panalytical Ltd., Royston, UK), and the result is shown in Figure 1a. This is in line with the particle size distribution between 15 and 45 µm reported by the powder supplier. The shape of the powder particles was determined by scanning electron microscopy (SEM, Philips XL30, Philips Electron Optics, Hillsboro, OR, USA) to be predominantly spherical (Figure 1b).

Selective laser melting was performed on an SLM 125 (SLM Solutions). Based on preliminary single-track experiments and building of several sets of trial samples, the parameters presented in Table 2 were selected for processing final specimens. These parameters enable reasonably good production efficiency. The build platform was preheated to 200 °C. The processing was performed under argon atmosphere with an oxygen level of <0.1%. A stripe pattern scanning strategy with 7 mm stripe length was applied. There was a 66° rotation in scanning direction between the successive layers. For comparison, a sample was also processed with all similar parameters (Table 2) but with no rotation in the scanning direction between the layers. Two kinds of specimens were built, 10 mm × 10 mm × 10 mm cubes for microstructural examination and corrosion testing, and vertical cylindrical samples with 11 mm diameter and 75 mm height for the tensile test specimens.

The density of the built samples was measured by X-ray Computed Tomography (CT) scanning with a Phoenix V|tome|x S industrial high-resolution CT and X-ray system (Baker Hughes, Houston, TX, USA). CT scanning allows three-dimensional evaluation of the total volume of internal defects in the sample volume. The density of the samples was also determined from polished cross-sections using optical microscopy, by measuring the fraction of porosity by image analysis using Image-J software.

Post-processing heat treatments for the as-built samples were performed in Modu Temp (XRF Scientific/Modutemp Pty Ltd., Perth, Australia) laboratory furnaces. Four different annealing temperatures were applied, i.e., 950, 1000, 1050, and 1100 °C. Holding times were 5 min and 60 min (the latter only applied at temperatures 1000 and 1050 °C), followed by water quenching. 

The microstructure of the specimens in the as-built state and after different heat treatments was examined: Specimens were cut in half in vertical (building) direction, mounted in resin, and ground and polished up to 0.4 µm surface finish with colloidal silica. The microstructure was characterized with scanning electron microscopy using FEI Nova Nano SEM 200 equipment (Waltham, MA, USA), including an Oxford NordlysMax^2^ EBSD Detector.

Microhardness of the specimens was measured with a Future-Tech FV-700 Vickers hardness tester (Holbrook, NY, USA), using test load of 1 kg and dwell time of 10 s. The measurements were performed on cross-sections polished to 0.4 µm finish. Ten replicates were performed on each specimen for reproducibility.

**Figure 2:**
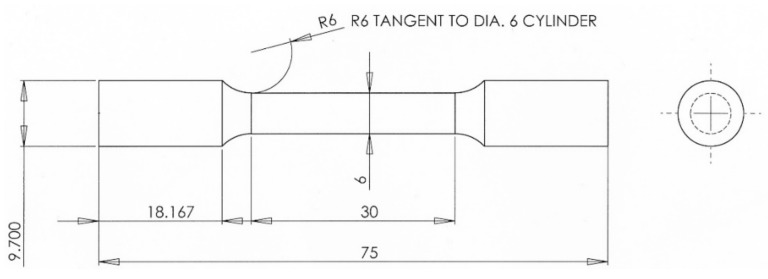
Tensile test specimen geometry. All dimensions are in millimeters.

Mechanical properties of the samples were determined with an Instron 50.2 kN tensile tester. Three specimens were tested for each material condition. The geometry of the tensile test specimens used is presented in Figure 2. The specimens were machined out of vertically-built cylindrical samples, i.e., the tensile testing direction was along the build direction.

Residual stress distribution along a middle height cross-section of cylindrical specimens, both in the as-built condition and after heat treatment (1000 °C/5 min), was measured using the contour method. The contour method is a destructive method for full cross-sectional mapping of residual stresses, in which the specimen is carefully cut in two and the deviation of the surface contour of the cut plane from planarity, due to relaxation of residual stresses normal to the cut surface, is measured and used to calculate the original residual stresses [31]. Samples were sectioned using electric discharge machining (EDM). The sample was attached to the machine with clamps on both sides to prevent movement during sectioning. The speed of the wire was 1.3 mm/min and the diameter of the wire was 0.25 mm. Before sectioning, the sample surface was coated with a conductive glue to avoid possible cutting-induced artefacts at the surface. The surface profile measurement was performed using a white-light interferometer (WLI) Bruker Contour-GT (Billerica, MA, USA). WLI is a non-contact optical method used for measuring discontinuous surface profiles with high precision, depth resolution being in the order of a few nanometers. Using WLI provides high spatial resolution and enables residual stress contour measurement from relatively small samples, such as the AM samples used here. The measurement data were denoised with a Kolmogorov–Zurbenko filter [32], with filtering parameters adjusted manually to provide optimal trade-off between retaining spatial resolution and avoiding noise-induced artefacts in the measurement. The residual stresses were computed from the measured surface displacements using Elmer open-source FEM-code. 

Corrosion resistance of the SLM-processed materials was studied by cyclic potentiodynamic polarization (CPP) testing. Three different types of specimens were studied: SLM-processed as-built material, SLM-processed heat-treated (1000 °C/5 min) material, and a conventional cold-rolled and annealed duplex stainless steel 2205. Electrochemical testing was performed at room temperature using a potentiostat Interface 1010E (Gamry Instruments, Warminster, PA, USA). A three-electrode electrochemical cell containing 300 mL of neutral 0.6 M NaCl electrolyte was used with an exposed area of 1 cm^2^. Duplex stainless steel specimens were the working electrode, saturated calomel electrode (SCE) as reference electrode, and Pt-wire as counter electrode, respectively. CPP curves were recorded at a scan rate of 1 mV s^−1^, after 1 h stabilization at open circuit potential (OCP). The scan range was set as follows: Start at 300 mV below OCP (cathodic potential) and the direction of polarization was reversed at 1.0 V_SCE_. Two replicates were performed for each sample to ensure the reproducibility. The tendency to pitting corrosion was estimated based on the characteristics of the CPP curves. 

## 3. Results and Discussion

**Figure 3:**
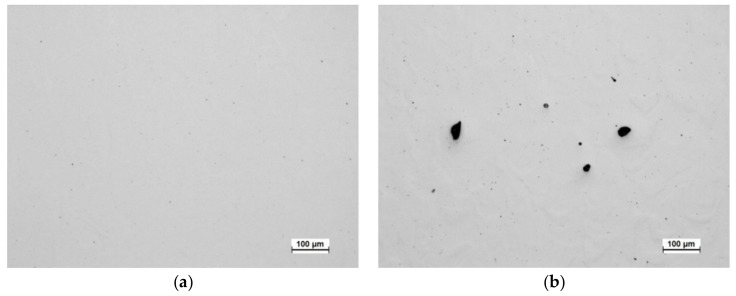
Optical micrographs of (**a**) the as-built sample processed with 66° rotation in scanning direction and (**b**) the as-built sample processed without rotation in scanning direction between the layers.

Preliminary tests for establishing suitable SLM processing parameters for 2205 duplex stainless steel suggest that a sufficiently high laser power, 250 W, should be used to minimize porosity. The process parameters used, with the 66° rotation in the scanning direction between the successive layers, resulted in a density of 99.97%, determined through image analysis of polished cross-sections from optical microscopy (Figure 3a), and of 99.99% according to X-ray CT analysis. The density value obtained with CT was higher, probably due to the limited resolution of the method, not detecting porosity smaller than ~30 µm with the used measurement parameters [33]. The density of the non-rotated scanning strategy sample was lower, at 99.01%, and there were notably larger pores present (Figure 3b). It is evident that to minimize porosity, it is beneficial to use a scanning strategy with rotation in the scanning direction between the layers. However, according to a recent comprehensive study on AISI 316L stainless steel, laser power plays a more dominant role in porosity formation during SLM processing than the laser scanning pattern [11].

**Figure 4:**
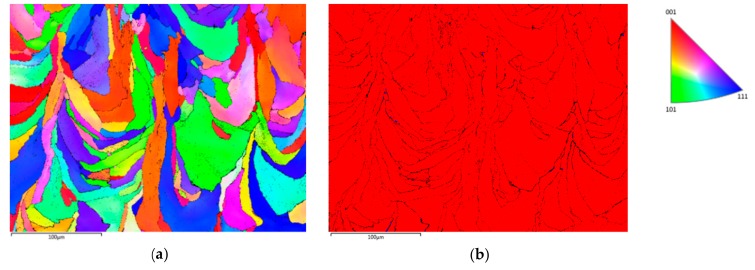

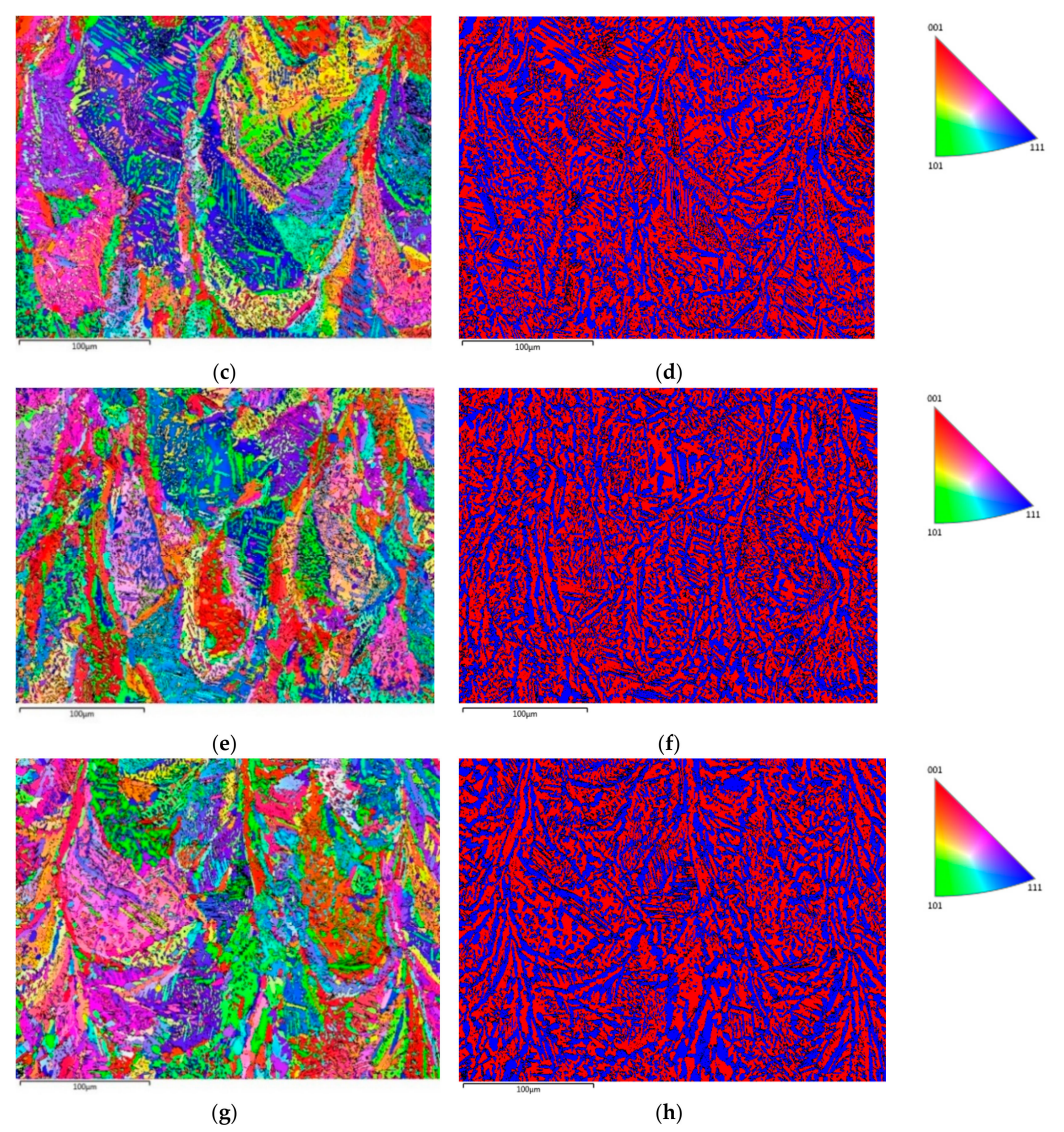
EBSD inverse pole figures (**a**,**c**,**e**,**g**) and phase maps (**b**,**d**,**f**,**h**) of the as-built specimen with 66° scanning rotation (**a**,**b**), specimen heat treated at 1000 °C/5 min (**c**,**d**), specimen heat treated at 1050 °C/5 min (**e**,**f**), and specimen heat treated at 1000 °C/60 min (**g**,**h**). Ferrite phase is colored with red and austenite phase with blue in the phase maps.

Microstructural features of the as-built and three heat-treated specimens, namely 1000 °C/5 min, 1050 °C/5 min, and 1000 °C/60 min, are illustrated in Figure 4 through EBSD inverse pole figures and phase maps. The as-built material (Figure 4a,b) exhibits a columnar grain morphology oriented along the build direction (vertical axis). Such an as-solidified grain morphology with columnar grains spreading over several successive layers along the building direction, as a result of epitaxial growth, is often observed in AM-processed metals [34]. The volume fraction of ferrite is approximately 99%. Minor austenite nucleation has occurred at the ferrite grain boundaries. Such a nearly fully ferritic solidification microstructure of as-built selectively laser-melted duplex stainless steels, due to high cooling rate, is consistent with the earlier results [6,7]. When the as-built stainless steel was heat treated, significant fractions of austenite phase were observed, nucleating at both melt pool boundaries and inside the ferrite grains. Figure 4c,d shows the microstructure after annealing at 1000 °C/5 min, Figure 4e,f after annealing at 1050 °C/5 min, and Figure 4g,h after annealing at 1000 °C/60 min, respectively. The driving force for the recrystallization and austenite nucleation during post-processing heat treatments is the high dislocation density of as-built stainless steel [7].

**Figure 5:**
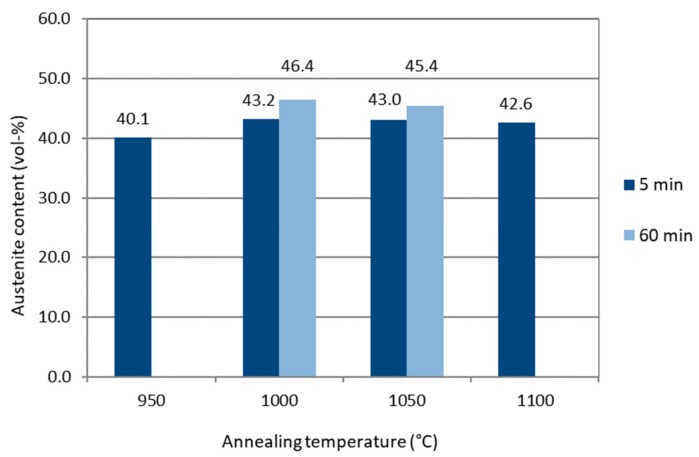
Austenite content (vol.%) for the various heat-treated specimens.

Volume fractions of the austenite phase, estimated from the EBSD data, are presented in Figure 5. With a 5 min annealing time, the austenite content is above 40% regardless of the annealing temperature (between 950–1100 °C) and reaches a maximum level of 43.0–43.2 vol.% at 1000 °C and 1050 °C annealing temperatures. For a longer 60 min annealing time, the austenite volume fraction is increased further, up to 46.4%. Several studies have shown that annealing at 1000–1050 °C results in the highest austenite content in duplex stainless steels [7,15,35,36]. Annealing reactivates the transformation of the metastable ferrite into austenite, which was suppressed by fast cooling. With increasing the annealing temperature, more metastable ferrite transforms into austenite, but simultaneously thermodynamic equilibrium favors the transformation of austenite into ferrite [35]. A balance is reached in annealing at temperatures between 1000 °C and 1050 °C.

Duplex stainless steels can be prone to intermetallic phase precipitation at high temperatures. Sigma phase precipitation can drastically deteriorate toughness and corrosion properties [37]. Sigma phase forms at temperatures between 650 °C and 950 °C, and chi phase below 800 °C. These phases may form during slow cooling through this temperature region. To avoid the sigma and chi phases, the heat treatments in this study were performed at 950 °C and higher temperatures, followed by rapid quenching in water. Presence of sigma phase was not detected in the microstructural examinations.

Microhardness results, mean values of ten measurements, are presented in Table 3. For reference, the microhardness of conventional cold-rolled and annealed duplex stainless steel 2205 was also measured, under similar experimental conditions. In the as-built condition, the microhardness is the highest, 337 HV1. After different heat treatments, the microhardness is reduced, with a greater reduction at the higher annealing temperatures and longer holding times. Even after annealing, the hardness remains higher compared to the cold-rolled duplex stainless steel of similar chemical composition.

The results of the tensile tests are presented in Table 4. Three specimens were tested for each condition and the results presented are the mean values. For comparison, typical values of tensile properties of the conventionally produced, cold-rolled, and annealed duplex stainless steel 2205 are also presented. In the as-built condition, the material has high yield and tensile strength but low elongation to fracture. This is consistent with [7], where this behavior is explained by the observed high dislocation density and nitride precipitates formed during rapid solidification of the nitrogen supersaturated ferrite matrix. Annealing decreases the strength, but significantly increases the ductility. The yield and tensile strength values of the SLM post-processing heat-treated specimens are still markedly higher than those of the cold-rolled and annealed reference alloy 2205. Especially the ultimate tensile strength is considerably higher, in the range of 812–869 MPa, for the SLM-processed materials after heat treatment. Equally high levels of yield and tensile strength have been observed for powder metallurgically-produced duplex stainless steels in the annealed condition, due to the considerably small phase size of the microstructure [38]. 

The mechanical properties obtained in this study are better than reported in [7] for the same duplex stainless steel 2205; the higher strength is probably due to the lower level of porosity and the considerably higher ductility (28% [7] vs. 46% elongation to fracture after annealing at 1000 °C/5 min) due to higher austenite volume fraction (34% [7] vs. 43%). AM-processed stainless steels often exhibit higher yield and tensile strength and hardness, but lower ductility, when compared to their traditionally processed counterparts [13]. However, both superior strength and ductility of SLM-processed stainless steel AISI 316L are also reported [11].

**Figure 6:**
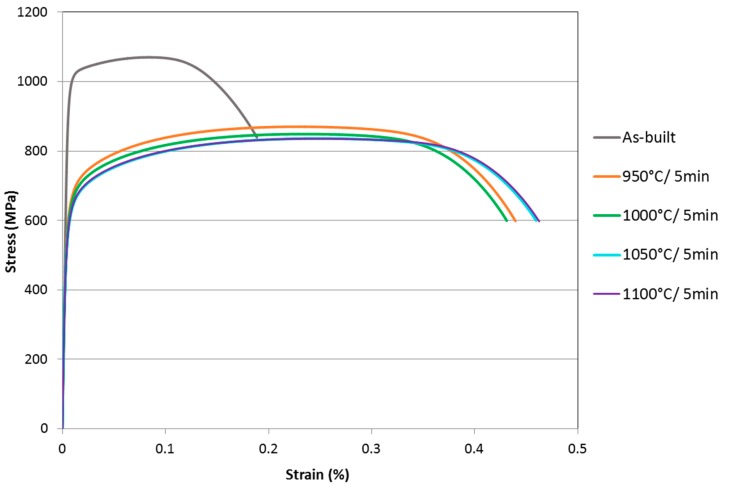
Engineering stress-strain curves for 2205 duplex stainless steel in as-built and annealed conditions.

Engineering stress-strain curves measured for the different specimens are presented in Figure 6. The as-built condition, being almost fully ferritic and presumably having very high dislocation density like determined in [7], shows the highest yield strength and ultimate tensile strength in comparison to the heat-treated conditions.

**Figure 7:**
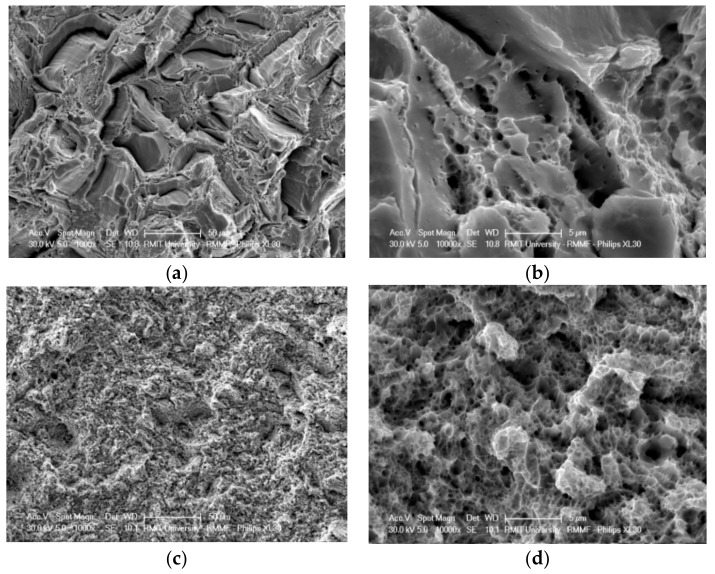
Fracture surfaces of tensile test specimens: (**a**,**b**) As-built and (**c**,**d**) heat treated 1000 °C/5 min.

Images of fracture surfaces of tensile test specimens, as observed by SEM, are presented in Figure 7. In the as-built condition (a,b), when the microstructure is 99% ferritic, the fracture surface contains both brittle intergranular and ductile dimpled features. In the post-processing heat-treated conditions, when the microstructure consists of ferrite and austenite, the fracture surfaces consist of very small dimples (c,d), indicating a fully ductile fracture. The two phases have different deformation modes, and austenite improves the ductility and toughness of the material. The fracture surface features are quite similar in all the heat-treated specimens, demonstrating a fully ductile fracture, regardless of the annealing temperature or the holding time. This observation is in accordance with the tensile test data (Table 4 and Figure 6) and the EBSD results (Figure 4d,f,h), which show relatively similar mechanical behavior and microstructural features, such as the volume fractions and spatial distribution of the two phases, between the variously heat-treated specimens. 

**Figure 8:**
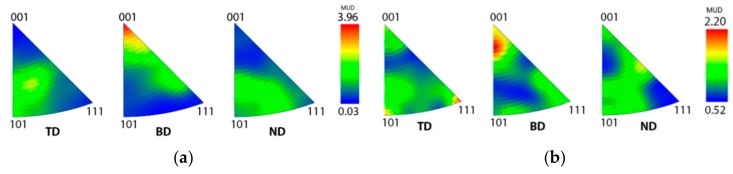
Pole figures of the body-centered cubic (BCC) ferrite phase in the as-built samples with different scanning strategies: (**a**) No scanning direction rotation between layers and (**b**) scanning direction rotated 66° between layers. TD = transverse to building direction, BD = building direction, and ND = normal to building direction.

In SLM, the scanning strategy applied influences the resulting microstructure. In the present study, two samples were produced for investigating the effect of scanning rotation, both processed with otherwise similar parameters, but the first with a 66° rotation in scanning direction between the successive layers and the other one with no rotation between scan layers. According to the EBSD results, there were no clearly dominant preferred crystallographic orientations in the as-built microstructure processed with a 66° rotated scanning strategy (Figure 4a). Inverse pole figures of the BCC ferrite phase in the two as-built samples with different scanning strategies are presented in Figure 8. The maximum intensity of the preferred crystallographic orientations is higher and there is stronger <001> alignment in the building direction in the sample processed without scanning direction rotation (Figure 8a), in comparison to the sample with the 66° scanning direction rotation between the layers (Figure 8b). Therefore, to decrease the texture and anisotropy in mechanical properties, it seems beneficial to use the rotated scanning strategy. 

For selective laser melting of other metal alloys, it has been reported for Ti-6Al-4V that a 67° rotation scanning strategy is favorable in terms of enhanced ductility, in comparison to 90° rotation, due to the formation of a more equiaxed microstructure in the building direction [39]. For Ni-25 at.%Mo alloys, it has been shown that a rot-scan strategy, where the scanning direction is rotated by 67° between each layer, produces a microstructure where the melt pools in the cross-section appearing in irregular half ellipse shapes, with a different morphology in each layer [40]. The mismatch in the position of the melt pool in each layer influences the development of the crystalline texture. 

**Figure 9:**
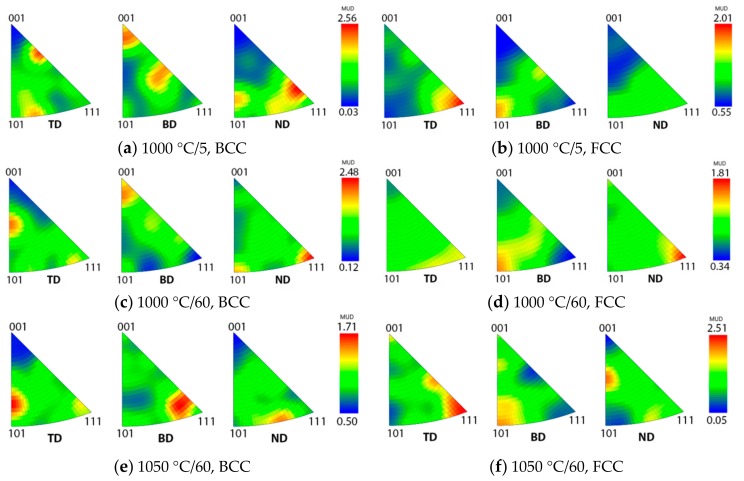
Pole figures of BCC (ferrite) and face-centered cubic (FCC; austenite) phases of heat-treated specimens: (**a**,**b**) 1000 °C/5 min, (**c**,**d**) 1000 °C/60 min, and (**e**,**f**) 1050 °C/60 min.

After a post-SLM processing heat treatment, the strength of the crystallographic texture in SLM-processed metals can be considerably reduced [41]. Inverse pole figures of selected heat-treated specimens are presented in Figure 9. Crystallographic texture is more intense in the ferrite phase than in the austenite. The texture of the ferrite phase is somewhat weaker after the heat treatments, especially after 60 min annealing time (Figure 9c), in comparison to the as-built samples.

**Figure 10:**
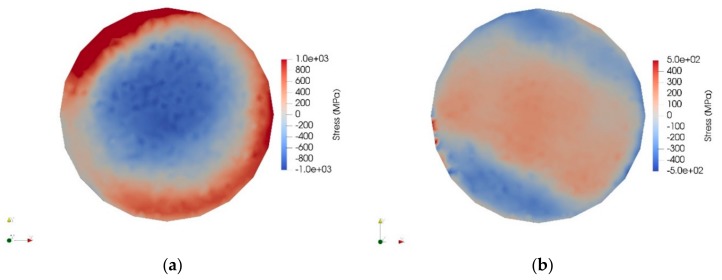
Residual stress distribution in a cross-section of (**a**) the as-built specimen and (**b**) the heat-treated (1000 °C/5 min) specimen.

According to the residual stress measurements, in the as-built material, the residual stress state on the side surfaces of the cylindrical samples is tensile, whereas most areas inside the specimen are under compression (Figure 10a). Because of constrained horizontal contraction, the deposition introduces vertical tensile residual stresses along the side surfaces and compressive residual stresses in the inner part [23,42]. The maximum level of the tensile residual stress is 800 MPa, which is similar to the measured strength values of the as-built material (Table 4). After heat treatment (1000 °C/5 min), the residual stress distribution is considerably different (Figure 10b): The outer surface layers show compressive residual stresses, balanced by tensile stresses in the inner parts of the specimen. The magnitude of the residual stresses is lower in the heat-treated state, and the compressive stress state in the outer surfaces is beneficial. 

**Figure 11:**
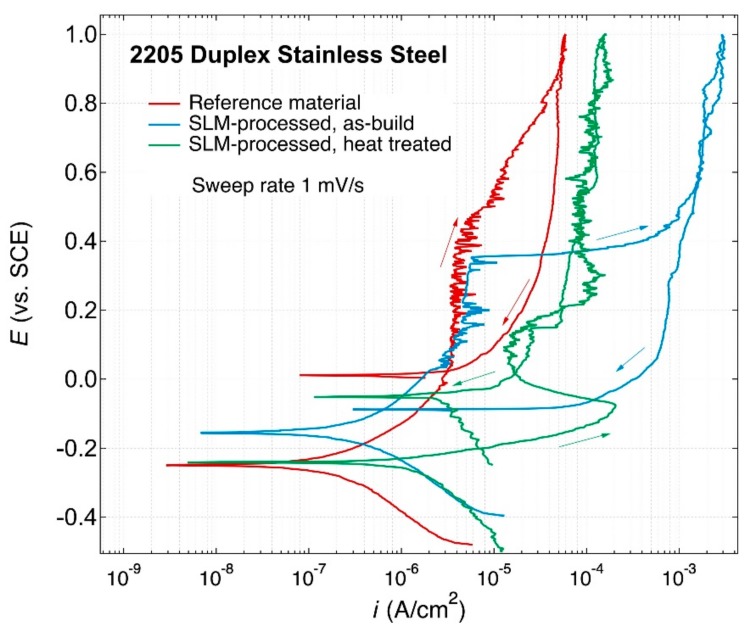
Cyclic potentiodynamic polarization curves of as-received cold-rolled and annealed reference alloy 2205, as-SLM-processed DSS 2205 and SLM-processed DSS 2205 with post-heat treatment in neutral 0.6 M NaCl solution at room temperature, respectively. The arrows indicate the polarization direction.

Results of corrosion testing are shown in Figure 11. During the cyclic potentiodynamic polarization in the potential range from 300 mV below OCP up to 1.0 V_SCE_, pitting breakdown potential (*E*_pit_) of 0.4 V_SCE_ was observed for the SLM-processed as-built material. Marked hysteresis was observed when the direction of polarization was reversed and the return polarization curve followed an active path. The re-passivation potential of the SLM-processed as-built material was low, and the reduced re-passivation potential may be related to the porosity of the material. Pitting corrosion resistance of the SLM-processed material was significantly improved by the heat treatment, based on the pitting breakdown potential and hysteresis. After the heat treatment of the SLM-processed material, similarly to the conventionally produced reference 2205 material, no distinct pitting initiation and hysteresis of the cyclic polarization curves in the studied potential range were observed, in contrary to the SLM as-built sample. Both of these samples have austenitic-ferritic microstructure, contrary to the as-built SLM sample. The conventionally produced DSS 2205 and the SLM-processed as-built material show a lower passive current density than the heat-treated SLM-processed material. Reasons for this require further investigation.

The as-built SLM-processed material was almost fully (99%) ferritic, whereas after the heat treatments the austenite content increased to 40–46%. In general, duplex stainless steels exhibit greater corrosion resistance than single-phase austenitic and ferritic stainless steels in chloride-rich media [43]. This is predominantly due to the high alloy content of Cr and Mo. Recent research has indicated that the galvanic interaction between the two phases favors the enhancement of the passive film by modifications of its chemical composition and semi-conductive properties [44].

## 4. Conclusions

High-density duplex stainless steel 2205 specimens have been successfully produced by selective laser melting. With an optimized combination of processing parameters, porosity of the material was <0.03% and excellent mechanical properties were achieved. The as-built microstructure was almost fully ferritic, but the desired dual-phase microstructure was produced by post-processing heat treatment. After annealing at temperatures from 950 °C to 1100 °C, over 40 vol.% of austenite phase was formed. Annealing decreased the high yield and tensile strength of the as-built material, but significantly increased the ductility. The microhardness (255–280 HV1), yield strength (520–560 MPa), and tensile strength (810–870 MPa) of the SLM-processed and heat-treated specimens were markedly higher than those of the conventionally-produced reference alloy 2205 with similar chemical composition, while the uniform elongation was in a similar range (23–25%). The scanning strategy with 66° rotation in the scanning direction between the layers decreased the porosity and resulted in less pronounced crystallographic texture and anisotropy. Residual stress measurement showed that post-processing heat treatment, being essential for establishing the dual-phase microstructure, was also very beneficial for reducing the residual stresses in the material and, more importantly, changing the residual stress state of the surface layers from tensile to compressive. Electrochemical cyclic potentiodynamic polarization testing in 0.6 M NaCl solution at room temperature showed that the heat treatment improved the pitting corrosion resistance of the as-built SLM-processed 2205 duplex stainless steel.

## Figures and Tables

**Table 1:** Chemical composition of the used SAF 2205 powder (wt.%).

Element	Cr	Ni	Mo	Mn	N	Si	C	P	S	Fe
wt.%	22–23	5–6	2.8–3.6	2.0 max	0.15–0.21	1.0 max.	0.03 max.	0.03 max.	0.015 max.	bal.

**Table 2:** Optimal selective laser melting (SLM)-processing parameters determined through the production pre-trials.

Laser Power (W)	Scan Speed (mm/s)	Layer Thickness (µm)	Hatch Spacing (µm)	Track Energy (J/mm)	Laser Energy Density (J/mm^3^)
250	850	50	100	0.29	59

**Table 3:** Microhardness (HV1) and its standard deviation of the SLM duplex stainless steel for different annealing treatments. Cold-rolled and annealed reference alloy 2205 is included for comparison.

Sample	As-Built	Annealed 950/5	Annealed 1000/5	Annealed 1050/5	Annealed 1100/5	Annealed 1000/60	Annealed 1050/60	Ref. Alloy 2205 *
Hardness (HV1)	336.6 ± 6.4	279.9 ± 10.3	279.8 ± 11.2	270.5 ± 8.8	255.2 ± 4.2	262.6 ± 9.6	265.0 ± 8.5	247.0 ± 1.5

* Cold-rolled and annealed duplex stainless steel 2205.

**Table 4:** Tensile mechanical properties of the test materials, with standard deviation of the results.

Sample	As-Built	Annealed 950°/5	Annealed 1000°/5	Annealed 1050°/5	Annealed 1100°/5	Annealed 1000°/60	Annealed 1050°/60	Ref. Alloy 2205 *
Yield strength (MPa)	950.0 ± 9.2	560.8 ± 3.3	549.0 ± 4.9	535.5 ± 9.2	524.0 ± 2.8	531.7 ± 2.5	517.1 ± 2.9	450–500
Tensile strength (MPa)	1071.3 ± 6.9	868.7 ± 2.3	848.0 ± 1.0	836.7 ± 1.1	813.0 ± 0.6	824.4 ± 1.8	812.1 ± 4.1	600–655
Uniform elongation (%)	7.0 ± 1.3	22.9 ± 0.4	23.9 ± 1.2	24.4 ± 1.1	24.1 ± 0.4	25.2 ± 0.9	25.3 ± 0.9	>25
Elongation to fracture (%)	16.0 ± 1.1	39.7 ± 1.6	45.8 ± 2.6	42.1 ± 3.5	39.7 ± 3.4	43.4 ± 2.5	41.8 ± 1.5	-

* Typical reference values from stainless steel producers.
